# Women's reproductive health in the Sakha Republic (Yakutia)

**DOI:** 10.3402/ijch.v73.25872

**Published:** 2014-10-30

**Authors:** Natalia I. Douglas, Tatiana U. Pavlova, Tatiana E. Burtseva, Yana G. Rad, Palmira G. Petrova, Jon Ø. Odland

**Affiliations:** 1 Department of obstetrics and gynecology, North-Eastern Federal University, Yakutsk, Russia; 2 The Yakutia research center for Complex medical problems, Yakutsk, Russia; 3 Department of Community Medicine, Faculty of Health Sciences, The Arctic University of Norway, Tromsø, Norway

## Table of Contents

Introduction

Chapter 1. Female reproductive health

 1.1. Regional variations in female reproductive health in the Russian Federation

 1.2. Female reproductive health in Yakutia

Chapter 2. Materials and methods

Chapter 3. The physical and reproductive health of study participants

Chapter 4. Microscopic and bacteriologic studies of vaginal and cervical canal secretions

Chapter 5. Hormone studies

Chapter 6. PELVIC ultrasonography

Chapter 7. Induced abortion in Yakutia and potential solutions

 7.1. Trends in induced abortion in Yakutia

 7.2. Reproductive health in teenage girls and women of reproductive age following induced abortion

Conclusions

References

## Introduction

According to the Russian National Population Census from October 9, 2002, the resident population of Yakutia was 949,300. Of the total population, 365,200 were of Yakut ethnicity. This means that Yakutia failed to retain its status as a region of more than 1 million residents. In the previous census, from 1989, the population was 1,094,100. This population decline is associated with a large rate of emigration, as well as a decrease in the rate of natural increase. In 2002, the total fertility rate was 2.53 births per woman in rural areas and 1.56 in urban areas, compared with the figure necessary for population replacement – 2.23 births per woman. In rural areas, the total fertility rate remained sufficient for replacement of parents in the population by their children. In Russia as a whole, the total fertility rate was 1.32 births per woman. A number of regions in Central Russia had an even lower total fertility rate – 1.1 births per woman. In difficult economic times, the reproductive health of the indigenous Northern peoples, including men, and particularly in Yakutia, is cause for legitimate concern.

Moskvina found ethnicity-associated variations in prenatal development for women living in Yakutia (1). Through the course of the entire pregnancy, Yakuts and Evenks consistently showed significantly decreased foetal growth parameters compared with Caucasians. Currently, there is no data on the impact of different climates and regional conditions on reproductive development and function in teenage girls and women of reproductive age. Nor is there ethnicity-specific data. A discussion about health promotion and resolving the demographic crisis must take into consideration both the realities of the situation (available resources, regional characteristics, reproductive trends, etc.) and the population's values system. At the top of the priority list is improving the health of teenage girls and women of reproductive age, a task which requires a multifaceted approach (2).

Unwanted pregnancy and subsequent abortions are significant problems, especially in the Russian Federation. Of particular concern is the extent to which teenage girls are affected – greater than 10% of all abortions are performed on women younger than 19 years of age (3). Changes in a teenager's environment and socio-economic situation certainly have an impact on reproductive health. Many studies have been carried out on the reproductive health of both teenage girls and women, taking into account region-specific variations (4–6). One topic that has not yet been examined in the indigenous peoples of Yakutia is reproductive health following the termination of a teenage pregnancy or the termination of a woman's first pregnancy.

## Chapter 1. Female reproductive health

### 1.1. Regional variations in female reproductive health in the Russian Federation

The demographic situation in the Russian Federation is at a critical point. This stems from, primarily, a high mortality rate among those of working age (16.1 deaths per 1,000 population in 2008), and an extremely low birth rate. For population replacement, the total fertility rate should be at least 2.14 births per woman; in 2004 it was 1.34 (7). According to *Demoscope Weekly*, the number of permanent residents of the Russian Federation fell by 0.15% between January and October 2007 (8). In Russia as a whole, 1.3 times more people died than were born. In 8 regions of the Russian Federation, this figure was significantly more, between 2 and 2.5. If the birth rate remains unchanged, each succeeding generation of Russians will comprise only 60% of the total of the previous generation (9).

Along with this established pattern of decreased reproduction, evidenced by a steep decline in the fertility rate and a low rate of second births, is an increase in married couples experiencing fertility problems. Between 15 and 17% of married couples in Russia are infertile. A rate of 15% is considered a critical figure, requiring government intervention (10). Approximately 5 million Russian women of reproductive age are in need of treatment for infertility, and this figure increases every year (11–13). Further, childlessness is a problem that affects both partners. According to Korneeva, in 62% of cases of infertility, the problem rests with the woman; in 36.7% of cases, it lies with both partners (14).

The demographic situation is accompanied by the declining health status of the Russian population as a whole, including men (15). Kulakov and others designated the state of Russian reproductive health as a national security issue (16). According to Radzinsky and Semyatov, the reproductive and physical health of women in Russia has, over the past 10 years, substantially deteriorated (17). The number of girls in the population who are between 15 and 17 years of age, considered the future reproductive reserve of the nation, comprise only 4.7% of the female population, and a mere 8.2% of the population of women of reproductive age, 15–49 (18).

The prevalence of epidemics such as alcoholism, drug addiction and HIV/AIDS also has a negative impact on demographic indicators. Poverty and low socio-economic status are known risk factors for drug and tobacco abuse (19). Chasnoff and others found that 33% of pregnant women use alcohol and narcotics (20). Twenty-one percent of women admitted to using alcohol up until they discovered they were pregnant, and 11% of pregnant women continued using alcohol even after they knew that they were pregnant.

Various authors found that alcohol use among pregnant women in Moscow was widespread – 47% consume alcohol in some form or another. Prior to pregnancy, 9.4% of the women were categorized as heavy drinkers. Of these women, all older than 31, 67% continued to consume alcohol during pregnancy, and 53% reported halting their consumption of alcohol during the first trimester of pregnancy (21, 22).

While abuse of nicotine, psychostimulants and hallucinogenic substances with psychological dependence does not lead to disruption of the menstrual cycle in teenage girls (23), sporadic use of opiates with psychological dependence, however, can lead to secondary amenorrhoea.

In terms of HIV, the Russian Federation remains one of the most hard-hit regions of Eastern Europe. Over the past 10 years, the number of new cases of HIV has increased 27 times, and among women, 49 times. New HIV diagnoses in pregnant women has increased 190 times from 0.6 per 100,000 tested in 1996 to 110.0 in 2006 (24).

According to the Program of Action of the United Nations’ International Conference on Population and Development (1994), “reproductive health is a state of complete physical, mental and social well-being and not merely the absence of disease or infirmity, in all manners relating to the reproductive system and to its functions and processes. Reproductive health therefore implies that people are able to have a satisfying and safe sex life and they have the capability to reproduce and the freedom to decide if, when and how often to do so” (25).

- A woman's reproductive health is an indicator of her overall health, and must therefore be preserved (26). Only a healthy mother can give birth to a healthy child and only a healthy child can become a healthy mother or father. It is here that the task of producing healthy offspring and preserving the reproductive health of women exits the medical realm and becomes a societal issue, with its solution at the government level. Reproductive functions are the oldest and most robust of our functions, yet the female reproductive system is particularly sensitive to the effects of harmful environmental and lifestyle factors (27, 28). Complications during the perinatal period put the child at a higher risk for physical and infectious conditions, which in turn can cause reproductive problems.
- Based on the results of physical examinations performed on workers from more than 20 sectors of manufacturing and agriculture, as well as women living in highly polluted regions (approximately 250,000 women and 50,000 newborns), several recommendations were made for the practice of obstetrics and gynaecology, and environmental reproductive health (29, 30):
- A woman's reproductive system is very sensitive to the effects of unfavourable conditions of any origin, and of any intensity, including subthreshold.
- The appearance of environmentally induced reproductive pathologies involves specific and non-specific reactions. The latter is found to a significantly greater extent.
- Environmentally dependent disruptions of the female reproductive system can have hormonal, biochemical and immunologic symptoms. In most cases, they have a similar presentation and resemble one another under the influence of a variety of anthropogenic factors.
- Clinical disturbances include an increase in menstrual disruptions, non-specific chronic diseases of the reproductive organs, hyperplastic processes, decline in fertility, increase in pregnancy and delivery complications, decline in foetal and newborn health and an increase in miscarriages.
- The extent of an environment's negative impact on a woman, and her tolerance to adverse conditions, is determined by a woman's phenotype, age, occupation, living conditions, as well as the amount and type of exposure to the deleterious agent.
- Environmentally dependent changes in the female reproductive tract develop in 3 phases. The health of the foetus and the newborn depends on which phase of adaptation to environmental aggressors the body was in at the onset of pregnancy, and for the majority of its duration.

The role of the endometrium is often overlooked when studying birth and reproduction, despite the fact that the endometrium plays a key role in the initiation and support of a successful pregnancy. Both immunologic and psychological stresses play an important role in the functioning of the endometrium, presumably through inflammation (31).

In addition to environmental (32) and socio-economic factors, neuroendocrine disturbances also play a role, as well as inflammatory processes of the internal genitalia.

Medical factors which play a decisive role in population growth through influences over fertility and mortality include: abortions, births to HIV-positive mothers, prevalence of HIV, spousal infertility, miscarriage, infant death, death from cancers of the female reproductive tract and maternal death (33).

The state of womens’ reproductive health can be characterized by region-specific features and is closely dependent on the environmental and demographic situation in the region (34, 35). In the past decade, in various regions of the Russian Federation, several studies have been conducted on establishing and maintaining a healthy reproductive system in teenage girls. Data show a decline in the health of young women, an increase in gynaecologic disorders, abnormalities in physical and reproductive development and changes in sexual and reproductive behaviour. The state of health of girls and young women today does not inspire optimism, with an increasing morbidity rate from gynaecologic diseases in girls (33). According to the Ministry of Health of the Russian Federation, the overall morbidity rate for children and teenagers has increased. Between 65 and 70% of teenage girls suffered from some form of chronic condition and 112 per 1,000 girls had a disorder of the reproductive tract (18, 36). Among girls in Russia, the prevalence of gynaecologic disease is high, between 12 and 15% on average (37). According to data collected through routine physical examinations in 2003–2004, 29% of girls between the ages of 1and 15 had some sort of disturbance of the reproductive tract (38). This means that, in the first decade of the 21st century, Russian women entering their most crucial reproductive years will by and large have problems with their reproductive organs.

In Moscow city, the decline in reproductive health in girls and young women of Moscow takes place against the background of decreasing birth rates, increasing in mortality rate, insufficient material wealth, as well as irresponsible reproductive and contraceptive behaviours on the part of teenagers (39). Similar trends are being observed in Moscow Oblast (40). Furthermore, the incidence of infectious or inflammatory diseases of the reproductive organs has also increased.

Other regional studies demonstrate common trends throughout the Russian Federation (41–46).

Studies conducted in Yamalo-Nenetsky Autonomous Okrug have shown that in the coming decades the number of teenage girls will decrease by more than 20%, a fact that in and of itself will contribute to depopulation trends and threaten national security in Western Siberia. In addition to this, the reproductive development of girls in this region has been shown to be slower than average. Such a trend is occurring along with a high rate of abnormalities in physical development and poor physical health due to widespread environment-related illnesses affecting the urinary and gastrointestinal tracts, as well as anaemia. There is also a high prevalence of gynaecologic disease (about 40%) (47, 48).

In northern Tyumensk, development of the reproductive system is subject to stressors specific to that region (49). Indicators of physical and reproductive health are inversely proportional to the number of generations a family has resided in the region. There are other region-specific factors affecting reproductive health: higher values for chest circumference, a decrease in the average size of the pelvic inlet, an “intensification of sexual development,” leading to a shorter period of early puberty. Women living for a long period of time in the Far North have a certain “Northern” metabolism along with diminished antioxidant protection, associated with a progressive deficiency in fat-soluble vitamins due to low intake and the high energy demands from chronic environmental stress (50). An increase in the rate of anaemia from 757 per 1,000 births in 1993 to 976 in 2002 has been noted, together with an increase in urinary tract disorders among pregnant women of the Far North, from 580 per 1,000 births in 1993 to 632 in 2002 (51).

Yankovskaya studied the impact of seasonal changes on the outcomes of pregnancy in women of the Kola Peninsula: the best outcomes were found in babies conceived between November and April, when the nights are longer (52).

It is also important to examine the implications of early pregnancy losses. The term “pregnancy loss” encompasses maternal and perinatal mortality, as well as abortions and ectopic pregnancies (53).

In the Russian Federation in 2006, 387 women died of complications from pregnancy or labour. Maternal mortality is an important criterion for evaluating the quality of obstetric–gynaecologic services, as well as in understanding the state of public health, and motherhood and childhood services in a country. In its report of the 18th FIGO World Congress of Obstetrics and Gynaecology in 2006, the World Health Organization called maternal mortality the “quiet epidemic.” In terms of maternal mortality, Russia is considered to be in the “uncivilized country” category (54, 55). According to the Russian Ministry of Health and Social Development, maternal mortality has decreased by 31%, from 38 per 100,000 live births in 2002 to 24 in 2007 (56). The maternal mortality rate differs significantly in different regions of the Russian Federation. In the Northwestern Federal Okrug, the maternal mortality rate is 19 per 100,000 live births, while in the Ural Federal Okrug it is 34 per 100,000 live births. The Far Eastern Federal Okrug maintains a consistently high maternal mortality rate (34 per 100,000 live births in 2005 and 32 in 2006) (57). The leading cause of maternal mortality in the Russian Federation is from complications during pregnancy, birth and the post-partum period (74%). Next is death caused by complications from an abortion (20%), followed by ectopic pregnancy (58). Overall, in Russia, 33% of maternal mortalities are preventable. In developed countries maternal mortality is mostly attributable to causes such as thromboembolism, non-gynaecologic infections and complications from anaesthesia (59). Maternal mortality in Khabarovsky Kray is at a moderate level, and is decreasing. From 1993 to 2002, the maternal mortality rate decreased from 97 to 62 per 100,000 newborns. Khabarovsky Kray is a large region with low population density and an unfavourable climate and environment. The primary causes of maternal mortality are complications from abortion and toxaemia of pregnancy (60). There are, however, significant differences in the rates and causes of perinatal death among the different regions of the Russian Federation (61, 62).

Back in 1928, Professor V.S. Gruzdev called the declining birth rate a “fight against motherhood” and showed that it is manifested in different ways, predominantly through artificial termination of pregnancy. After more than 80 years, induced pregnancy termination continues to be a reason women are able to remain voluntarily childless (63).

Despite an overall decrease in the absolute number of abortions in the past decade, abortion is the second most common cause of maternal mortality. In 2005, 38 per 1,000 women of reproductive age underwent abortion in the Russian Federation (64). One of the contributing factors is the fact that induced abortion is the only method of birth control covered by the compulsory medical insurance system (65). Indeed, in Russia, the use of effective methods of contraception is a third of that in economically developed countries. A notable achievement has been reducing the abortion-to-birth ratio to 1:1. However, this decrease in abortions did not occur as a result of a decrease in the termination of first pregnancies (66). Within 6 months of their first sexual encounter, between 28 and 46% of young women terminate a pregnancy through surgical abortion (67). In the United States, however, the abortion rate between 1996 and 2003 was 8%, and in China it was 21% (68).

Gazazyan and Khardikov, in their article “Why abortion remains a popular birth control method in Russia,” noted that the main reason for the continued high rate of abortions is that abortion has been firmly entrenched in the Russian contraceptive tradition, especially as it is practically and economically accessible (69).

Currently, Russian women have a number of options for contraception available to them. However, a large number of couples ignore the notion of “safe sex,” without acknowledging the significance of post-abortion complications (63). According to Sinchikhin, in Russia a mere 25–40% of women of reproductive age use modern methods of contraception, while 60–75% of women place themselves at risk for an unwanted pregnancy (70).

The United States Center for Disease Control and Prevention analyzed data on the sexual and reproductive health of persons between the ages of 10 and 24 for the period 2002–2007 (71). They found that the pregnancy rate for Hispanics and Blacks between the ages of 15 and 19 was significantly higher (133 and 128 per 1,000 population), than among their non-Hispanic White peers (45 per 1,000 population).

Abortion in youth is a serious problem and a major cause of reproductive health problems. The phenomenon of teenage pregnancy, young motherhood, the rejection of the institution of family by modern youth and sexual freedom are factors that will have an impact on the quality of future generations. Pregnancy in a young woman is, as a rule, unplanned 50–60% of the time, and, in 30–40% of cases, unwanted, leading to the high abortion rate (72). In 2004, nearly 1 in 10 abortions in Russia was performed on a teenager (18). This could result in the trend towards childless marriages and feto-maternal disease due to inflammation caused by disruption of the endometrium and failure of implantation of the blastocyst (73, 74).

In a study of the course of teenage girls’ pregnancies following termination of a first pregnancy, there was a risk of miscarriage in 1 out of 2 pregnancies, toxaemia of pregnancy in 63%, and anaemia in 74% of women (75). The risk of foetal abnormalities was found to increase by 2.8 times if the second pregnancy was initiated fewer than 6 months after termination of the first (76). Various studies have found a high rate of abortions across Russia, and it appears to be increasing in rural areas also (77, 78).

The question remains of how to minimize complications from induced abortions. A partial answer to this question, from a reproductive standpoint, has been found in the research of Litvak and Apresyan. They found that1of the reasons that fertility does not return following laparoscopic procedures to restore flow through the fallopian tubes is an immunologic anomaly, diagnosed by measuring embryotropic autoantibodies (79, 80). After reviewing more than 300 publications from the past 20 years, Gleicher concluded that without consideration of a woman's immune system, it is impossible to accurately evaluate the state of her reproductive health (81), and there exist a variety of biochemical and immunological tests to identify differentiation and morphogenic factors that play a direct role in the development of the embryo and foetus (82–84) as well as a woman's reproductive potential, her ability to maintain a pregnancy, and give birth to a healthy child (85). Optimal levels of regulatory autoantibodies are essential for the normal functioning of a woman's body, including her reproductive functions. Opportunities now exist for developing new methods for diagnosis and prognosis, and for precise immunocorrection of disturbed reproductive function (82, 86).

The level of production of embryotropic autoantibodies is significantly altered under the influence of various harmful environmental factors (chemical, physical and biological agents). Immunologic disturbances are the earliest sign of destructive changes in the body that are of environmental origin (87). Indeed, almost all teratogens and embryocydic factors act to alter the activity of the various components of the immune system.

Immunoreactivity as evaluated by the embryotropic autoantibody ELI-P test allows us not only to predict a non-developing pregnancy but also to carry out pathogen-specific therapy to prevent pregnancy loss (88):

- Normoreactive patients exhibit and are treated for an endocrine pathology.
- Hyporeactive patients exhibit persistent infections, for which they are treated with anti-inflammatory agents and immunomodulators and enzymatic therapy, treatment aimed at boosting immune function.
- Hyperreactive patients present with concomitant acute/subacute infectious processes and undergo antimicrobial or antiviral therapy. They also undergo treatment for concomitant gynaecologic and non-gynaecologic infections.

A persistent inflammatory process arising from a chronic autoimmune condition leaves a woman immunosuppressed, resulting in diminished production of embryotropic antibodies (hyporeactivity) (89, 90). This hyporeactivity is maintained for a long period of time following surgical treatment for gynaecologic conditions (up to 6 years) in women who did not undergo therapy during the post-operative period (91).

### 1.2. Female reproductive health in Yakutia

A bleak demographic situation can be observed in the Sakha Republic (Yakutia). The 2002 census showed that the republic failed to maintain its status as a region with greater than 1 million residents. In 2002, the fertility rate in urban areas was 1.56 births per woman, compared to 2.23 necessary for population replacement. The fertility rate in rural areas was 2.53 births per woman. In Russia as a whole, the fertility rate was 1.32 births per woman, including several regions in Central Russia with a fertility rate hovering around 1.1 births per woman. In 2010, the population of Yakutia was 949,347, including 490,218 females. Of the females, 26,470 were of reproductive age, 21,731 were teenagers and 100,939, young girls.

In Yakutia, obstetric–gynaecologic services are provided by 38 birth departments housed in a variety of hospitals – this includes 516 beds for mothers and newborns, 268 beds for high-risk pregnancies and 500 gynaecologic beds.

In 2011, according to the regional Medical and Preventive Facility, there were 16,193 births in Yakutia. Premature births constituted 3.3% of all births. Uncomplicated births comprised 46.5% of all births. In 2011, 6 girls under the age of 14 gave birth. In 2010, 1 girl under the age of 14 gave birth. There were 7,522 single births. There were 150 multiple births, of which 148 were twins.

Non-gynaecologic illnesses had a large impact on the period of gestation, birth outcomes, complications and illnesses in newborns. While the overall health of pregnant women has improved in Yakutia, indicators from 2011 nevertheless are significantly worse than in the Russian Federation as a whole or the Far Eastern Federal Okrug. The types and frequency of health problems are shown in Table 1.1.

**Table 1.1:** Proportion of pregnant women carried to term with health problems, Yakutia, 2007–2011

	Anaemia	Kidney disorders	Circulatory disorders	Thyroid disorders	Late toxaemia
2007	51.0	32.5	7.7	19.5	14.9
2008	45.5	28.5	7.9	18.2	13.0
2009	44.0	30.3	8.1	19.6	13.2
2010	44.8	25.5	9.4	18.1	12.8
2011	43.7	30.1	8.5	16.7	13.2
Far Eastern Federal Okrug 2010	33.9	18.5	11.3	8.8	19.5
Russian Federation 2010	34.7	19.2	10.4	6.2	18.1

Preventive measures have been undertaken in an effort to decrease complications from pregnancy. These include dispensing anaemia treatment, micronutrients, iodine and other compounds free-of-charge to those with a maternity certificate. About 76% of women were reached early in pregnancy for intervention, while 78% of women were seen by a physician during the first 12 weeks of pregnancy.

Between 2007 and 2011, the mean annual maternal mortality rate was 19 per 100,000 live births, compared to 17 per 100,000 live births in the Russian Federation as a whole in 2010. All cases of maternal mortality underwent autopsy and were discussed at clinical–pathological conferences with the participation of relevant parties from Yakutsk and the regions.

During this period, the mean annual perinatal mortality rate was 8.6 per 1,000 births. Among the most important causes are antenatal hypoxia and asphyxiation during delivery, respiratory distress and congenital anomalies.

In terms of morbidity of newborns, the frequency of diagnoses is shown in Table 1.2.

**Table 1.2:** Morbidity in newborns, Yakutia, 2007–2011

Cases per 1,000 live births	2007	2008	2009	2010	2011	Russian Federation 2010
Overall morbidity (born ill and became ill)	322.5	310.5	305.1	282.4	284.9	354.9
Antenatal hypoxia, asphyxia during delivery	170.3	150.9	123.8	99.8	88.6	98.1
Infectious diseases, specific to the perinatal period	19.7	17.3	12.6	10.4	11.4	16.4
Newborn bacterial sepsis	0.2	0	0.1	0.1	0.1	0.2
Poor growth and malnutrition	39.4	48.4	43.5	39.4	46.7	87.0
Neonatal jaundice due to haemolysis, or due to other and unspecified causes	42.4	30.9	37.4	36.8	40.8	77.1
Congenital anomalies	54.1	57.0	65.5	65.0	75.4	30.0
Respiratory distress syndrome	33.7	35.4	34.6	35.1	32.7	41.2
Trauma from delivery	13.7	12.9	10.6	10.6	10.3	31.2
Haemolytic disease of the newborn	2.7	2.7	2.9	3.4	2.0	8.6

In 2011, 48 out of 1,000 in the female population suffered from some gynaecologic illnesses in Yakutia. The leading gynaecologic disorders include pelvic inflammatory disease (53%), menstrual disturbances (12%), erosion and ectropion of the cervix (12%), endometriosis (3%) and infertility (2%).

The rate of malignant neoplasms of the female reproductive tract has increased by 9% between 2007 and 2011. The mean annual incidence rate of various cancers during this period is: breast 37 per 100,000, cervix 19 per 100,000, ovary 10 per 100,000 and uterus 9 per 100,000.

The trends in abortions are shown in Table 1.3.

**Table 1.3:** Trends in abortion, Yakutia, 2007–2011

Year	Total abortions	Abortions per 1,000 women of reproductive age	Ratio of births-to-abortions
2007	14,090	51.1	1: 0.9
2008	13,120	48.0	1: 0.86
2009	12,059	44.6	1: 0.76
2010	10,848	40.7	1: 0.68
2011	9,900	37.2	1: 0.61

Because data from official medical statistics are dependent on the population's willingness to seek medical attention, a more in-depth picture of the reproductive health of the population of Yakutia must take several factors into consideration: ethnic origin, place of origin, accessibility of medical services. This study was conducted with that goal in mind.

## Chapter 2. Materials and methods

We have chosen 3 primary topics to study: identifying characteristics and determining factors of reproductive health and behaviour in teenage girls and women of reproductive age; determination of regional-, ethnicity- and age-related factors affecting and influencing reproduction in the teenage years; implementation and evaluation of interventions designed to improve the reproductive health of teenage girls and women of reproductive age, and decrease reproductive losses in Yakutia.

This study was carried out at health care and educational institutions in the city of Yakutsk, the villages of Sayylyk (Ust-Yansky region), Olenek (Allaikhovsky region), Berezovka (Srednekolymsky region), Andryushkino and Kolymskoe (Nizhnekolymsky region). A total of 578 women were studied, including 476 from the indigenous Yakuts and Evenks.

A group of 318 teenage girls was recruited while presenting for routine physical examinations in organized groups. These girls were 8–11th graders from schools in the city of Yakutsk and the study villages. First- and second year students from Yakutsk State University also participated. The study participants were divided into 3 groups:

Group I: 102 participants of European origin, born and living in Yakutia, consisting of 50 young girls and teenage girls, and 52 women of reproductive age;

Group II: 296 participants of Yakut ethnicity, consisting of 186 young girls and teenage girls, and 110 women of reproductive age; and

Group III: 180 participants of Evenk ethnicity, consisting of 82 girls and teenage girls, and 98 women of reproductive age.

The study consisted of the following components:

- Clinical examination and questionnaire survey (n=578).
- Ultrasonography (n=3,311).
- Hormonal studies – levels of sex and gonadotropic hormones (n=1,178).
- Immunoreactivity studies based on embryotropic autoantibodies (n=160).
- Microscopic studies of smear specimens (n=2,109).
- Molecular-genetic studies (PCR) of vaginal and cervical canal secretions (n=240).

The patient information questionnaire included basic demographic information and relevant medical and social history, with special interest in the following fields:

- Timing of menarche, menstrual function and regularity and length of menstrual cycles.
- Sexual history: age of first sexual encounter, any official marriages.
- Use of contraception: type and duration of use, any complications.
- History of gynaecologic infections, duration, outcome.
- History of surgical procedures: type (laparoscopy or laparotomy) extent, and recovery period.
- Fertility: parity, intergenerational interval, information about prior pregnancies (outcome for mother and child, surgical procedures); if history of in-vitro fertilization then number of attempts, protocols, complications, outcomes, reasons for lack of success.

The physical examination was carried out in a clinic setting at room temperature. A variety of anthropometric indicators were recorded, including height and weight, body mass index, sitting height, chest circumference, shoulder width, leg length (from the greater trochanter to the floor) and pelvic dimensions. Sexual development and menstrual function were evaluated, including inspection of body hair and presence of galactorrhoea.

The stage of sexual development was determined based on the Tanner Method (92), as modified by Skorodok (93). Pelvic dimensions were measured with a gynaecologic pelvimeter, using standardized methods. Non-gynaecologic disorders were identified using a specially developed procedure combining clinical, laboratory and functional studies.

With their consent, an additional gynaecologic examination was performed on girls with a history of sexual activity. In the presence of specific complaints and with consent, girls with no history of sexual activity underwent recto-abdominal examination of the pelvis.

All patients underwent ultrasound using the ALOKA-1700 with a 3.5 MHz abdominal transducer and a 6.5 MHz vaginal transducer. They also were examined using the Voluson – 730 Pro V using the IC5-9H∣GIN and RAB2-5∣OB transducers.

The following were examined: the cervix, endocervical thickness and homogeneity, the position, size and shape of the uterus and the state of the endo- and myometrium. Identification and diagnosis of the following conditions was emphasized: endometrial hyperplasia, chronic endometritis, adenomyosis, uterine fibroids. Participants were evaluated for signs of inflammation of the uterine adnexa and presence of adhesive processes in the pelvis. The ovaries were also examined for size, position and presence or absence of folliculogenesis. Folliculogenesis was evaluated by ultrasound monitoring of the number and size of follicles, as well as the thickness of the endometrium in all studied menstrual cycles.

Ovarian reserve was measured by counting the number of antral follicles smaller than 10 mm in diameter using ultrasonography on day 2–3 of the menstrual cycle. Depending on the number of follicles, participants were categorized as having:

- Normal ovarian reserve: 6–10 follicles in both ovaries.
- Poor ovarian reserve: 5 or fewer follicles in both ovaries.
- Multifollicular ovaries: more than 10 follicles in both ovaries.

Serum hormone levels, including prolactin (PRL), luteinizing hormone (LH), follicle-stimulating hormone (FSH), thyroxine, triiodothyronine, thyroid-stimulating hormone (TSH), estradiol (E2), progesterone, cortisol and testosterone were measured using immunofluorescence.

The ELI-P-Test-1 (ELISA-detected Probability of Pathology) was used to study serum immunoreactivity, as represented by the quantity and affinity of natural embryotropic autoantibodies working with protein regulators of embryogenesis. The ELI-P-Test-1 is based on a standard enzyme-linked immunosorbent assay test. Antibodies were identified with the following antigens:

- Myelin basic protein (antigen-1): the main protein in myelin, participates in regulating maturation of nerve fibres and haematopoetic cells.
- S-100 protein (antigen-2): a protein, which participates in regulating the migration of neuroblasts of the spinal cord and cerebral cortex, and their functional differentiation.
- Nuclear chromatin protein ACBP-14/18 (antigen-3): an acidic protein, closely associated with chromatin, involved in regulation of gene expression.
- MP-65 (antigen-4): a membrane protein, a member of the integrin superfamily of proteins, participates in regulation of intercellular adhesion.

The ELI-P-Test was used as a diagnostic tool. The tests were carried out according to the kit's instructions, approved by the Ministry of Health of the Russian Federation (94). The following evaluation criteria were used (95):

- Normoreactivity: serum reaction intensity with any of the antigens comprising 5–40% of the reaction of the reference serum.
- Hyperreactivity: serum reaction intensity with any of the antigens comprising 41% or more of the intensity of the reference serum.
- Hyporeactivity: serum reaction intensity with any of the antigens is below normal.

Specimens for bacterial culture were taken from the urethra, the cervical canal and the posterior vaginal wall. The specimens were collected with a fluted probe following preliminary massage of the urethra. From the posterior vaginal wall, discharge was collected using a spatula. The samples were then smeared on a glass slide, dried, and dyed with gram stain and examined under a light microscope. Table 2.1 describes the classification of the results.

**Table 2.1:** Microscopic evaluation of vaginal micro flora

Normal vaginal flora	Lactobacilli predominate; lack of gram-negative flora, spores, mycelia, pseudo hyphae, fewer than 30 leukocytes in the visual field, epithelial cells	Typical state of normal vaginal micro flora
Bacterial vaginosis (vaginal disbacteriosis)	Few or no lactobacilli, a large amount of bacteria with bacteroides, mobiluncus, and *Gardnerella vaginalis* predominating; identification of “key” cells among the upper layer of epithelial cells; few or no leukocytes in the microscopic field; few or immature phagocytes	Seen in women without clinical vaginitis
Vaginitis	Many leukocytes, macrophages, granular degeneration of epithelial cells, “mottled view,” activate phagocytosis, presence of opportunistic and pathogenic flora	Complains of white discharge, burning, itching

The procedure for collecting specimens for culture was as follows: contents of the cervical canal and the posterior vaginal wall were sampled with a sterile tampon. The samples were plated using a stroke technique over half of a Petri dish with 5% blood agar. After this, the tampon was immersed in a 1% sucrose solution. The cultures were incubated at 37°C and monitored daily. Upon the appearance of growth on the solid medium, colonies were inventoried for various morphologies, taking proportions into account. When the solution became cloudy, it was smeared on a glass slide (gram stain) and, depending on the microscopic findings, they were cultured on solid media (blood agar, vitelline-salt agar, endo agar). Next, visual identification and antibacterial sensitivities were carried out. The results were considered negative in the absence of growth on any solid media over 72 hours.

Identification of any DNA bacterial or viral infections was carried out by polymerized chain reaction (PCR). Collection, transport and storage of specimens was carried out in accordance with regulations for PCR diagnosis formulated by the Epidemiological Institute of the Ministry of Health of the Russian Federation in 2003. The following species were tested for: *Chlamydia trachomatis, Mycoplasma hominis, Ureaplasma urealiticum, Neisseria gonorrhoeae, Trichomonas vaginalis, Gardnerella vaginalis*.

Specimens were obtained from scrapings of the vulva, vagina and cervical canal. Extraction of clinical material was carried out with a container and transport media from AmpliSens. Material was extracted from the cervical canal. Mucus was extracted from the cervix using a tampon, a probe was inserted 1–1.5 cm into the cervical canal and rotated for 3–5 seconds. The probe was then removed, without touching the vaginal wall, and immersed in a sterile container with 0.3 mL of transport media.

STATISTIC 5.5 for Windows from StatSoft, Inc. was chosen for statistical analyses. In most cases, we presented our results in the format mean±standard error. The sample size was represented by the letter n.

## Chapter 3. The physical and reproductive health of study participants

For this study, 578 women were examined, including 102 women of Russian ethnicity born and raised in Yakutia. The other 476 women were indigenous Yakuts and Evenks (Table 3.1). There was no statistically significant difference in mean age between women of the different ethnic groups.

**Table 3.1:** Distribution by ethnicity, residence and age group of study participants

		Teenage girls		Women of reproductive age	
			
Ethnicity	Total	Number	Mean age	Number	Mean age
Russian	102	50	15.1±1.32	52	27.4±9.6
Urban	80	38		42	
Rural	22	12		10	
Yakut	296	186	14.8±1.23	110	26.9±7.6
Urban	172	111		61	
Rural	124	75		49	
Evenks	180	82	14.7±2.3	98	28.7±8.03
Urban	77	36		41	
Rural	103	46		57	

Greater than 3-quarters of the control group was found to live in an urban area. More than half of Yakuts were city dwellers, while more than half of the Evenks living in rural areas. However, no statistically significant difference between the Evenks and Yakuts was found.

Education level was recorded only among women of reproductive age, as all of the younger participants were attending either school or university (Table 3.2).

**Table 3.2:** Educational level of women of reproductive age

	Higher	Specialized secondary	Secondary
Russian	31 (59.6%)	19 (36.5%)	2 (3.8%)
Yakut	47 (42.7%)	52 (47.3%)	11 (10%)
Evenks	26 (26.5%)	33 (33.7%)	39 (39.8%)

More than half of the Russian women had attended an institution of higher education, significantly more than the Evenks. No statistically significant difference in level of education was seen between the Russian and the Yakut groups.

Respiratory and gastrointestinal disorders were the most common somatic ailments reported by teenage girls, afflicting 1 in every 3–4 girls (Table 3.3). More than 1 in 3 teenage girls among Russians were found to have respiratory problems, a significantly higher number than among the corresponding Yakut group. Evenk teenage girls experienced cardiovascular problem at a significantly higher rate than the other 2 groups.

**Table 3.3:** Proportion (%) of participants with non-gynaecologic disorders

		n	Cardiovascular	Respiratory	Gastrointestinal	Urinary	Thyroid
Russians	Teenage girls	50	10.0	36.0	34.0	18.0	6.0
	Women of reproductive age	52	17.3	17.3	26.9	26.9	21.1
Yakuts	Teenage girls	186	12.4	22.0	33.9	16.1	5.4
	Women of reproductive age	110	10.9	12.7	25.5	26.4	25.5
Evenks	Teenage girls	82	19.5	25.6	34.1	18.3	7.3
	Women of reproductive age	98	17.3	17.3	24.5	26.5	25.5

It should be noted that, in frequency of certain ailments, the rate among teenage girls exceeded that of women of reproductive age. Teenage girls experienced more respiratory problems (chronic bronchitis, chronic tonsillitis) in all 3 ethnic groups.

Some of the most crucial characteristics of the health of teenage girls are their physical development indicators, which are interconnected and interdependent with development of the reproductive system. Height and weight of all participants were measured (Table 3.4).

**Table 3.4:** Height and weight of study participants

Group		n	Height (cm)	Weight (kg)
Russians	Teenage girls	50	164.1±7.5	52.7±4.9
	Women of reproductive age	52	169.8±8.3	59.5±9.6
Yakuts	Teenage girls	186	153.1±6.0	50.2±7.0
	Women of reproductive age	110	160.7±6.3	56.2±8.2
Evenks	Teenage girls	82	152.0±7.5	49.2±11.3
	Women of reproductive age	98	159.1±5.6	61.6±10.6

Russian teenage girls and women of reproductive age of were found to be significantly taller in comparison to women from the study groups. On average, the greatest increase in height was found to occur at 13 years of age. In terms of underweight and overweight, 1 in 6 teenage Russian and Yakut girls were found to be overweight (Table 3.5). No statistically significant difference was found between rural and urban dwellers.

**Table 3.5:** Prevalence of underweight and overweight among study participants

Group		n	Underweight (%)	Overweight (%)
Russians	Urban	38	23.7	23.7
	Rural	12	16.7	16.7
Yakuts	Urban	111	25.2	17.1
	Rural	75	18.7	15.3
Evenks	Urban	36	13.9	8.3
	Rural	46	21.7	10.9

Somewhat more urban dwellers were found to be underweight; however, the difference between urban and rural dwellers was not statistically significant. A different situation was observed with young girls of Evenk origin – significantly fewer girls in both the urban and rural areas were overweight – approximately 1 in 10.

Physical development was found to be within normal parameters for most young Russian girls (84%) and the Yakut and Evenk groups (83%). Nearly 1 in 10 young Yakut girls (9%) exhibited delayed development, similar to what was observed among the Evenks or Russians. There was also no significant difference in precocious physical development between young girls from urban and rural areas. However, delayed physical development was observed more often in young girls residing in rural areas.

Pelvimetry revealed an increase in all 4 external pelvic measurements by the age of 10–11 years of age. At this age, all young girls of Yakut and Evenk origin began experiencing significant pelvic growth. The next increase in all transverse dimensions was observed at the beginning of the late pre-pubertal period. By 17 years of age, the overwhelming majority of young Yakut and Evenk girls (81%) ceased to experience pelvic growth, in contrast to 17-year-old girls from the Russian group. The average pelvic size was 0.5–1.2 cm smaller than Russian girls from the control group.

In examining development of secondary sex characteristics by age, we found that the level of sexual development was highly variable at age 13 (Table 3.6).

**Table 3.6:** Age range (years) and median for transition in stage of development of secondary sexual characteristics, young girls and teenage girls, by ethnic origin

	Russians		Yakuts		Evenks	
			
Formula	Age range	Median	Age range	Median	Age range	Median
Ma1P1A×1Me0	Up to 9–10	–	Up to 9–10	–	Up to 9–10	–
Ma2P1A×1Me0	10–14	11.5–12.5	10–14	11.5–12.5	10–14	11.5–12.5
Ma3P2A×1Me0	10–14	11.5–13.5	10–14	12.5–13.5	10–15	13.5–14.5
Ma4P3-4A×2Me1	11–16	12.5–14.5	11–15	13.5–14.5	12–16	13.5–14.5
Ma4P3-4A×3Me+	12–17	14.5–16.5	12–17	14.5–15.5	13–17	14.5–16.5
Ma5P4-5A×3Me+	13–17	16.5–17.5	13–17	16.5–17.5	14–17	16.5–17.5

No difference between groups was observed in the order of appearance of secondary sex characteristics. Breast development, on average, occurred at 11.4±0.6 years of age. The third Tanner stage of breast development, corresponding with menarche, appeared, on average, at age 13.5±0.6 for girls of all 3 groups.

The appearance of pubic and axillary hair was relatively uniform. The appearance of pubic hair began earlier in urban dwellers, 11.5±0.3 on average, than in rural dwellers, 12.5±0.5 years.

The growth of axillary hair also began earlier in urban dwellers – on average, 0.52±0.2 years earlier than in rural dwellers. However, both Yakut girls and Evenk girls were behind Russian girls in developing axillary hair by an average of 1 year.

Data from this study were compared to norms developed for Yakut girls (96, 97). We found that, in Yakut and Evenk girls exhibiting abnormal development before age 12, precocious development was observed, and then at age 13 and older – delayed development. By age 17, 25% of Yakuts and 21% of Evenks had not yet completed puberty, compared with 12% among the Russians. Rural dwellers had fewer girls who had completed puberty by age 17.

With a later onset of puberty, Yakut and Evenk girls experienced a faster increase in size of breasts. When examining the main anthropometric parameters, Yakuts and Evenks are similar to other indigenous peoples of the North, Siberia and the Far East. These groups are, in general, characterized by short stature and stockiness attributable to the harsh climate and other region-specific conditions.

An evaluation of menstrual function revealed that the age of menarche was on average 12–14.5 years of age (Table 3.7).

**Table 3.7:** Primary indicators of menstrual function

Group		n	Age of menarche, years	Duration of menstruation, days
Control	Teenage girls	50	12.6±1.1	4.3±0.9
	Women of reproductive age	52	12.8±1.6	4.8±2.9
Yakut	Teenage girls	186	13.8±0.99	4.9±2.1
	Women of reproductive	110	13.3±0.9	4.74±2.8
Evenks	Teenage girls	82	13.9±0.9	4.9±1.71
	Women of reproductive age	98	13.4±1.46	4.6±1.49

The average age of menarche in the Russian group was 12.6±1.1 years. In Yakuts, menarche was observed, on average, at 13.8±0.99 years of age, at an average height of 150.0±2.3 cm and an average weight of 42.1±2.8 kg. Evenks reached menarche at, on average, 13.9±0.9 years of age, at an average height of 149.1±3.1 cm and a weight of 43.4±1.7 kg.

Among the women, 33% had a menstrual cycle of fewer than 28 days; in 59% of the women, the menstrual cycle was 2–31 days, and it was greater than 31 days in 9% of the women.

The menstrual cycle became established either immediately or within the first 6 months among fewer than half of girls in the different ethnic groups. One-quarter of girls established regularity within 1year, while 1-quarter continued to experience irregular periods.

The prevalence of menstrual disturbances in young girls and teenage girls varied depending on age. Girls aged 12–14 experienced menstrual disorders significantly more often than girls aged 15–17 (72% compared to 27%). There was no statistically significant difference between ethnic groups.

Dysmenorrhoea was the most common cause of menstrual disorders (Table 3.8). More than half of girls exhibited dysmenorrhoea, with no difference observed between groups. Polymenorrhoea was diagnosed in 1 in 11 girls. Upon further analysis, it was found that polymenorrhoea was observed predominantly in girls with abnormal weight and height indicators. No statistically significant difference was found in frequency of polymenorrhoea between underweight and overweight girls. Along with dysmenorrhoea, hypomenstrual syndrome was also frequently seen among the indigenous peoples of Yakutia.

**Table 3.8:** Prevalence (%) of menstrual disturbances among study participants

		n	Dysmenorrhoea	Oligomenorrhoea	Polymenorrhoea	Hypomenstrual syndrome
Russian	Teenage girls	50	64	26	14	8
	Women of reproductive age	52	23.1	9.6	25	3.8
Yakut	Teenage girls	186	60.2	20.9	8.6	27.9
	Women of reproductive	110	20.9	9.1	22.7	11.8
Evenk	Teenage girls	82	57.3	36.6	8.5	29.2
	Women of reproductive age	98	21.4	25.5	26.5	16.3

These results show us that trends in physical and sexual development of girls from Yakutia are influenced by an array of unfavourable climatic and socio-economic factors acting during the pubertal period.

In the past decade, an increase in poor lifestyle habits has been observed in families with teenage girls primarily involving the consumption of alcohol or tobacco. According to the participants, either 1 or both parents abused alcohol in 1 out of 6 families (15%). One in 2 mothers smoked. Among teenage girls in the control group, 1 in 4 smoked. More girls from the indigenous groups smoked (32% among both Yakut and Evenk girls).

The average age at which the girls began to smoke was 14.0±1.02 years. Among the reasons for starting smoking, 80% of participants reported “for company,” 12.5% reported “it's cool” and 7.5% “to not stand out.”

When asked if they consume beer or other alcoholic beverages, 21% answered “yes.” Of participants who reported consuming alcohol, 12% reported consuming alcohol “frequently.” Of these, half of participants indicated that consumption of alcohol had “become a habit.” The average age for beginning to consume alcohol was 14.4±1.36 years. No statistically significant difference was observed between the different ethnic groups.

Three percent of teenage girls reported using drugs, 3-quarters of whom citing episodic use (“for company”), while the remainder said they “could not live without it.” The average age for beginning to use drugs was found to be 15.0±0.92 years.

Among teenage girls, 61% reported being sexually active. There was no statistically significant difference found between ethnic groups and place of residence. The average age of sexual debut was 16.05±1.07. A greater proportion of participants from rural areas reported having their first sexual experience before the age of 15, and this difference was statistically significant (63% vs. 32%). The current generation of teenage girls had a significantly earlier sexual debut compared to women of reproductive age.

The teenage girls were surveyed on their attitude towards sex and marriage. Saving sex for marriage was “desirable” among 10% of participants. Twenty percent of participants were against sex outside of marriage (62 participants, 19.5%). Eighteen years of age or older was considered to be the optimal age for sexual debut by 61% of participants.

Among sexually active teenage girls, 66% reported that their first sexual encounter was planned, 27% unplanned, and 7% were forced to some extent. About a third reported that their first sexual encounter occurred when they were under the influence of alcohol.

Table 3.9 summarizes the contraceptive behaviour of teenage girls and women of reproductive age. Methods of contraception such as barrier (condoms) and interruption of the sexual act were used by half of the participants, regardless of age or ethnic origin.

**Table 3.9:** Use of contraceptive method by study participants

Group		n	Barrier	Coitus interruptus	Rhythm method	Oral contraceptives	intrauterine device
Russians	Teenage girls	30	53.3	43.3	10.0	10.0	3.3
	Women of reproductive age	52	57.7	67.3	40.4	15.4	17.3
Yakuts	Teenage girls	114	56.1	44.7	9.6	8.8	7.0
	Women of reproductive age	110	46.4	74.5	51.8	14.5	15.5
Evenks	Teenage girls	49	46.9	42.8	10.2	6.1	6.1
	Women of reproductive age	98	52.0	76.5	52.0	3.1	8.2

Among sources of information about contraception and safe sex, less than 10% of teenage girls reported having discussed this with their mother or an older female relative. Approximately, half of teenage girls reported learning about contraception and safe sex from friends. Only 1 in 5 reported medical personnel or school as sources of information.

Among Russians and Yakuts, 1 in 3 women of reproductive age were married at the time of this study. This figure is significantly less than among Evenks, among whom more than half were married.

An evaluation of gynaecologic disorders among women of reproductive age showed that the most common disorders were benign disorders of the cervix and inflammatory processes of the uterus and adnexa (Table 3.10). Nearly half of participants were diagnosed with a benign disorder of the cervix, with no statistically significant difference between the groups. Infertility was diagnosed in 1 in 10 women, which is somewhat less than in the general Russian population.

**Table 3.10:** Prevalence (%) of gynaecologic disorders

Group		n	Benign disorders of the cervix	Inflammatory processes of the uterus and adnexa	Infertility	Hyperplastic disorders of the uterus	Ovarian dysfunction
Control	Teenage girls	30	23.3	13.3	–	–	33.3
	Women of reproductive age	52	44.2	21.1	13.5	17.3	9.6
Yakuts	Teenage girls	114	28.1	12.3	–	–	22.8
	Women of reproductive age	110	45.4	22.7	10.9	16.4	9.1
Evenks	Teenage girls	49	24.5	22.4	–	–	18.4
	Women of reproductive age	98	44.9	32.6	10.2	24.5	25.5

Evenks of reproductive age suffered from gynaecologic disorders significantly more often than Yakuts and Russians in the same age group.

Sexually active teenage girls experienced benign cervical disorders most often (1 in 4). Almost all cases of inflammation of the uterine adnexa were seen in 15- to 18-year-olds, apparently connected with an increase in sexual activity among teenage girls. Girls with no sexual experience predominantly presented with vulvitis and vulvovaginitis. There was no statistically significant difference among the ethnic groups.

Important for the demographic future of Yakutia are the reproductive goals of teenage girls. The great majority in the Russian group – 88% planned on having 1–2 children, 6% no children, and 6% more than 2 children. A statistically significant difference was observed in the indigenous groups, with 79% who planned on bearing 1 or 2 children, and 17% more than 2 children.

In terms of reproductive history (Table 3.11), significantly more women of reproductive age from the indigenous groups had a history of pregnancy than mong Russians. Overall, only 1 in 2 pregnancies resulted in a birth. Yakuts and Evenks had more early pregnancy terminations (induced abortion, spontaneous abortion, non-developing pregnancy). More than half of all pregnancies in the indigenous groups ended in induced abortion.

**Table 3.11:** Mean number of pregnancies, births and pregnancy losses among study participants

Group		n	All pregnancies	All births	Early pregnancy losses
Russians	Teenage girls	30	0.14+0.01	–	0.14+0.01
	Women of reproductive age	52	2.1±1.0	1.1±0.6	1.0±1.3
Yakuts	Teenage girls	114	0.28+0.08	0.12+0.01	0.16+0.07
	Women of reproductive age	110	3.9±2.1	1.7±1.2	2.2±1.3
Evenks	Teenage girls	49	0.31+0.06	0.10+0.01	0.21+0.05
	Women of reproductive age	98	4.2±2.19	2.1±1.0	2.5±1.1

Among teenage girls, 9% already had a history of pregnancy or were pregnant at the time of their examination. The average age of first pregnancy was 16.4±0.7 years. Among the girls who had undergone abortions, 54% reported receiving advice following the procedure – using oral contraceptives, and condoms in the absence of a regular sexual partner. However, only 29% reported following this advice. The reasons cited for not using oral contraceptives include “high cost” (45%), “necessity to take every day” (32%) and “forbidden by mother, sexual partner” (13%).

## Chapter 4. Microscopic and bacteriologic studies of vaginal and cervical canal secretions

Microscopic studies of vaginal and cervical canal secretions were carried out on all study participants. Four levels of vaginal hygiene was identified based on the presence of leukocytes in the secretions (Table 4.1).

**Table 4.1:** Degree of vaginal hygiene among sexually active teenage girls and virgins

			Degree of vaginal hygiene (%)			
			
Group		n	I	II	III	IV
Russians	Sexually active	30	27	30	13	30
	Virgins	20	40	35	5	20
Yakuts	Sexually active	114	18	35	21	26
	Virgins	72	42	36	8	14
Evenks	Sexually active	49	29	27	20	25
	Virgins	33	42	36	6	15

Level I is characterized by the presence of isolated leukocytes in the visual field, and a vaginal epithelium comprising small cells with round nuclei and a small amount of cytoplasm. The flora contains Doderlein's bacilli. Level II is characterized by a larger number of leukocytes in the visual field, a large number of epithelial cells with a less distinct shape, as well as cells from the various epithelial layers. A reduction in Doderlein's bacilli, as well as occasional pathological micro flora can be observed. In the third and fourth levels, a large number of leukocytes can be observed, with an abundance of different micro flora (predominantly cocci), abundant mucus and epithelial cells.

The distribution of the different levels is quite different among sexually active girls compared to virgins. Overall, vulvovaginitis was found significantly more often in girls with sexual experience than girls with no sexual experience. Vulvovaginitis was identified in just under half of sexually active girls, compared to 25% among virgins.

Results of microscopic studies of vaginal and cervical canal secretions of women of reproductive age are displayed in Table 4.2 (3).

**Table 4.2:** Degree of vaginal hygiene among women of reproductive age

		Degree (%) of vaginal cleanliness			
		
Group	n	I	II	III	IV
Russians	52	27	39	6	29
Yakuts	110	22	26	15	37
Evenks	98	18	25	24	34

Compared to Russian women, a lower proportion of indigenous Yakut and Evenk women were found to have normal vaginal flora. Vaginitis was diagnosed in one-third of Russian, compared to more than half of Yakut and Evenk women.

The most common microorganisms found were pathogenic microorganisms, especially *Candida albicans* and vaginal-associated pathogens (ureaplasma and mycoplasma). Gonorrhoea and trichomoniasis were found significantly less often compared with the other microorganisms (Table 4.3). The microbial landscape found in women of reproductive age did not differ appreciably from teenage girls.

**Table 4.3:** Distribution (%) of microorganisms identified in vaginal flora

Group		n	Chlamydia	Gonococci	Trichomonas	Candida albicans	Ureaplasma
Russians	Teenage girls	18	11.1	–	5.5	16.7	16.7
	Women of reproductive age	20	5	5	5	20	15
Yakuts	Teenage girls	70	11.4	4.3	7.1	14.3	14.3
	Women of reproductive age	57	7.7	–	7.7	19.3	17.5
Evenks	Teenage girls	29	10.3	6.9	6.9	20.7	20.7
	Women of reproductive age	56	10.7	3.6	5.4	17.8	12.5

In all cases of vaginitis, aggregates of microorganisms were identified, representing 2–3 types with equal or predominantly aerobic components. A preponderance of bacteria from the Enterobacteriaceae family were identified (*Escherichia coli* and *Enterobacter*). Cultures of vaginal secretions showed that teenage girls of all groups with vaginitis exhibited fewer lactobacilli than women of reproductive age (Table 4.4).

**Table 4.4:** Type of vaginal flora (%) among study participants with vaginitis

	Women of reproductive age			Teenage girls		
		
Microorganism	Russians (n=20)	Yakuts (n=57)	Evenks (n=56)	Russians (n=18)	Yakut (n=70)	Evenk (n=29)
*Lactobacillus*	55	39	39	33	31	31
*Corynebacterium*	–	–	7	–	–	–
*Enterococcus faecalis*	15	11	13	11	11	14
*Escherichia coli*	20	28	27	22	29	28
*Gardnerella*	15	12	13	11	11	14
*Staphylococcus epiderm*.	8	7	13	–	–	–
ß-haemolytic *Streptococcus*	8	7	11	11	10	10

## Chapter 5. Hormone studies

Participants’ hormone levels were measured in the early follicular phase.

In young girls, aged 10–14, significantly higher levels of LH and FSH were found in the Yakut and Evenk groups compared with the Russian group. In Yakut girls, the ratio of LH:FSH was 1: 1.5, while in Evenk girls, the ratio was 1: 2.1, both higher than the 1: 1 ratio among Russians. Comparison of these results with data on the development of secondary sex characteristics (Chapter 3) did not show the Yakut girls outpacing the Evenk girls. However, Evenk girls were found to have higher rates of oligomenorrhoea (37%) compared with Russian and Yakut girls (26 and 21%) (Table 5.1).

**Table 5.1:** Hormone levels during the early follicular phase among girls 10–18 years

Group	n	TSH (µIU/ml/)	T4 (ng/dL)	Cortisol (nmol/L)	Growth Hormone (ng/mL)	FSH (mIU/mL)	LH (mIU/mL)	Progesterone (ng/mL)	Estradiol (pg/mL)
Age 10–14									
Russians	20	6	1.17	90.38	1.08	3.07	3.05	1.89	68.9
Yakut	85	3.23	1.18	86.3	1.45	11.13	7.39	1.47	62.0
Evenk	44	5.41	1.17	92.1	0.54	14.47	6.95	1.7	56.9
Age 15–18									
Russians	30	3.76	1.07	295.31	1.08	4.17	3.84	2.89	125.7
Yakut	101	2.03	1.11	282.1	1.45	9.03	6.19	2.47	82.0
Evenk	38	3.41	1.12	273.6	1.49	10.13	5.06	2.7	71.9

Among teenage girls aged 15–18, the Yakut and Evenk groups exhibited higher levels of LH and FSH compared to the Russian group. The LH/FSH ratio in Yakut girls was 1: 1.46, 1: 1.2 among Evenk girls, and 1:1 among Russians. Girls in the 15–18 age group exhibited lower levels of LH and FSH in comparison to girls in the 10–14 age group; however, there was no change in the ratio of these hormones with age.

The levels of estradiol measured in the Yakut and Evenk groups were significantly lower than those in the Russian group. In comparing the hormone studies with physical development of the teenage girls, we found that at age 17, 1 in 4 Yakuts and 1 in 5 Evenks had not reached full sexual maturity, while this was seen in only 12% of Russian (Chapter 3). It is evident that the lower levels of estradiol and higher FSH and LH are part of a chain of events resulting in delay in sexual and physical development in girls from the indigenous populations of Yakutia.

In studying correlations between physical and sexual development data for girls 10–14 years of age, a distinct correlation was found between:

- Testosterone level and age, height and weight (r=0.53).
- Testosterone level and amount of hair growth in the axillary region (r=0.66) and pubic region (r=0.60).
- Testosterone level and level of breast development (r=0.50) and Tanner level of sexual development (r=0.56).
- LH level and age (r=0.51) and height (r=0.56).

According to the literature, the appearance of LH spikes during sleep demarcates the initiation of puberty (98). During the pre-pubertal and pubertal periods, girls secrete more FSH than boys, whose FSH secretion levels do not vary much between the pre-pubertal and pubertal periods. Oestrogen levels in girls are already higher than boys’ in the pre-pubertal period. According to the literature, testosterone levels in girls begin to increase during stage IV of development on the Tanner scale. Testosterone levels in girls remained low until they reach stage III of development on the Tanner scale (98, 99).

It is thought that adrenal cortical function is lower in indigenous children raised with the traditional way of life (100). We found significantly lower levels of cortisol in girls in the 10–14 age group compared with girls in the 15–18 age group. There was no statistically significant difference found between ethnic groups.

Hormone studies have shown that indigenous teenage girls living in their traditional homeland have better adaptive mechanisms for regulation. This is seen in increased pituitary function, regulating sexual maturation. Our results show high levels of the gonadotropic hormones LH and FSH in the indigenous groups.

In women of reproductive age, indigenous Yakut and Evenk women exhibited higher levels of TSH, and lower levels of free thyroxine compared with Russian women (Table 5.2). The difference in free thyroxine levels between the 2 groups was found to be statistically significant. These results can be explained by the presence of hypothyroidism in 1 out of 4 women in the indigenous population of Yakutia. Three-quarters of these cases were first diagnosed during this study. In the other cases, the women were already receiving the appropriate treatment.

**Table 5.2:** Hormone levels among women of reproductive age

Group	n	TSH (µIU/ml/)	T4 (ng/dL)	Cortisol (nmol/L)	Growth Hormone (ng/mL)	FSH (mIU/mL)	LH (mIU/mL)	Progesterone (ng/mL)
Russians	52	6.06	1.07	295.31	6.17	4.84	2.89	176.9
Yakut	110	7.86	0.81	282.1	9.53	7.19	2.47	182.0
Evenk	98	8.41	0.72	273.6	11.01	8.02	2.70	170.9

LH and FSH levels were significantly higher in Yakut and Evenk women in comparison to Russians. In women of reproductive age, the LH:FSH ratio was 1: 1.3; significantly lower than that seen in the young girls and teenage girls.

Yakutia is an area of high risk for disruptions to reproductive health. This is evidenced by the fact that 1 in 4 Yakut and Evenk women suffer from hypothyroidism, and 1 in 5 Russian women. There are also ethnicity-related differences in reproductive health, such as a shorter period between menarche and menopause in indigenous women, which is indicative of the unfavourable health environment in Yakutia.

## Chapter 6. PELVIC ultrasonography

Ultrasound studies were carried out on the pelvic organs during phases I and II of the menstrual cycle (Table 6.1). Results showed that the dimensions of the uterus (length, width, antero-posterior diameter) in indigenous teenage girls were significantly smaller than in girls of Russian ethnicity. These differences were found both in studies carried out on days 5–7 of the menstrual cycle, as well as on days 22–24. The length of the uterus on days 5–7 of the menstrual cycle was found to be 4.4±0.2 in the control group. Length was significantly less in the indigenous groups: 4.0±0.2 cm in the Yakut group and 3.8±0.2 cm in the Evenk group.

**Table 6.1:** Uterine ultrasound measurements (cm) at days 5–7 and days 22–24 of menstrual cycle

Group		n	Length	Width	Antero-posterior diameter	Endometrium
Days 5–7						
Russians	Teenage girls	50	4.4±0.2	3.9±0.2	3.1±0.2	1.9±0.2
	Women of reproductive age	52	4.9±0.7	4.4±0.3	4.3±0.9	3.4±1.2
Yakuts	Teenage girls	186	4.0±0.2	3.4±0.2	2.6±0.2	1.8±0.2
	Women of reproductive age	110	4.7±0.7	4.3±0.3	4.5±0.7	4.1±1.1
Evenks	Teenage girls	82	3.8±0.2	3.33±0.2	2.6±0.2	1.8±0.2
	Women of reproductive age	98	4.9±0.5	4.4±0.3	4.6±0.8	3.9±1.9
Days 22–24						
Russians	Teenage girls	50	4.5±0.2	4.5±0.2	3.5±0.2	4.4±1.8
	Women of reproductive age	52	4.9±0.7	4.8±0.5	4.4±0.9	7.4±2.2
Yakuts	Teenage girls	186	4.1±0.2	3.8±0.18	2.9±0.2	4.1±1.2
	Women of reproductive age	110	4.7±0.7	4.3±0.3	4.5±0.7	4.1±1.1
Evenks	Teenage girls	82	4.1±0.2	3.8±0.18	2.9±0.2	4.1±1.2
	Women of reproductive age	98	4.9±0.5	4.4±0.3	4.6±0.8	3.9±1.9

There was no statistically significant difference between groups in endometrial thickness. M-echo was used to evaluate the endometrium for form and uniform signal intensity. Endometrial thickness was found to be minimal in the first days following menstruation (phase I). The maximum thickness was found on days 21–23 of the menstrual cycle (phase II).

Ultrasound studies revealed significantly smaller ovarian size in young girls and teenage girls from the Yakut and Evenk group compared with the Russian group. Examination of the individual ovaries showed no significant difference in their development in participants, meaning that both ovaries were participating in the menstrual cycle during sexual development (Table 6.2). There was no statistically significant difference in uterine or ovarian size among women of reproductive age.

**Table 6.2:** Ovarian ultrasound measurements (cm) at days 5–7 of the menstrual cycle

			Right ovary			Left ovary		
				
Group		n	Length	Width	Thickness	Length	Width	Thickness
Russians	Teenage girls	50	3.3±0.3	2.5±0.3	2.0±0.2	3.3±0.3	2.7±0.3	2.0±0.2
	Women of reproductive age	52	3.4±0.3	2.8±0.3	2.8±0.2	3.6±0.3	2.8±0.3	2.6±0.2
Yakuts	Teenage girls	186	3.0±0.1	2.0±0.1	1.8±0.2	2.9±0.2	2.1±0.1	1.7±0.2
	Women of reproductive age	110	3.3±0.3	2.8±0.3	2.8±0.2	3.6±0.3	2.8±0.3	2.6±0.2
Evenks	Teenage girls	82	2.7±0.2	2.0±0.1	1.8±0.2	2.8±0.2	2.0±0.1	1.8±0.2
	Women of reproductive age	98	3.1±0.3	3.0±0.2	2.6±0.2	3.6±0.3	2.6±0.2	2.8±0.1

More detailed analysis of the data on the young girls and teenage girls showed that the uterus and ovaries both grew, on average, gradually and moderately. In girls of Russian origin, a prominent increase in the length, width and antero-posterior dimensions of the uterus was observed between the ages of 12 and 13 (increase in length – 1 cm, width – 1.4 cm, antero-posterior length – 1.1 cm). After this, growth proceeded more gradually, at a rate of 0.1–0.3 cm per year.

Uterine length was significantly less for girls in the indigenous groups compared with the Russian group. Statistically significant differences were observed among girls aged 10–11 and 12–16. A similar trend was observed with uterine length and antero-posterior diameter. By age 17, any statistically significant differences levelled out.

Among Russians, a prominent increase in ovarian length was observed between 11 and 12 years of age (by 0.6 cm). Thickness of the ovarian tissue was found to increase by 13 years of age (by 0.8 cm). No such prominent increase in growth was observed among Yakuts and Evenks (the maximum growth spurt was 0.4 cm). In girls younger than 17, ovarian size was significantly smaller in the Yakut and Evenk group compared with the Russian group. A high correlation was found between ovarian and uterine size and age of menarche (r=0.65).

It was found that, in participants under the age of 14, a majority of menstrual cycles were anovulatory (Table 6.3). In 15-year-olds, 3-quarters of menstrual cycles were anovulatory. By 16–17 years of age, ovulation began to be observed in half of the participants. At age 18, an overwhelming majority of menstrual cycles (80–87.5%) were ovulatory. No statistically significant difference in type of menstrual cycle was found between the ethnic groups.

**Table 6.3:** Menstrual cycles, by type, young girls and teenage girls

Group	Age	n	Anovulatory	Ovulatory
Russians	10–14	20	85.0	15.0
	15	8	75.0	25.0
	16	7	57.1	42.9
	17	7	42.9	57.1
	18	8	12.5	87.5
Yakuts	10–14	85	87.1	12.9
	15	21	76.2	23.8
	16	23	56.5	43.3
	17	26	46.1	53.8
	18	31	16.2	83.8
Evenks	10–14	44	84.1	15.9
	15	9	77.8	22.2
	16	10	60.0	40.0
	17	7	42.8	57.1
	18	10	20.0	80.0

Frequency of ovulation in teenage girls was found to increase gradually, reaching its maximum frequency during the sixth year of menstruation.

Through the course of our ultrasound studies, a number of sonographic findings were revealed (Table 6.4). Ultrasonographic signs of polycystic ovarian syndrome were observed significantly more often in the Yakut and Evenk teenage girls than in Russians. In women of reproductive age, ultrasonographic signs of polycystic ovarian syndrome were observed only in isolated instances. Ultrasonographic evidence of chronic salpingo-oophoritis and chronic endometritis was observed significantly more often in Evenks than in the other groups.

**Table 6.4:** Abnormal ultrasonographic findings (%)

Group		n	Ovarian cysts	Polycystic ovarian syndrome	Out-of-phase endometrial thickness	Chronic inflammation of uterus and adnexa	Uterine fibroids
Russians	Teenage girls	50	10	2	8	13	–
	Women of reproductive age	52	8	–	8	21	13
Yakuts	Teenage girls	186	10	9	28	12	–
	Women of reproductive age	110	10	2	16	23	13
Evenks	Teenage girls	82	10	7	29	22	–
	Women of reproductive age	98	8	1	18	32	22

Uterine fibroids were not identified among teenage participants. Among women of reproductive age, uterine fibroids were identified significantly more often in Evenks than in the Russian or Yakut groups. Out-of-phase endometrial thickness was found significantly more frequently in the indigenous women than in Russians. Teenage girls were more frequently found to have a “thin” endometrium.

## Chapter 7. Induced abortion in Yakutia and potential solutions

### 7.1. Trends in induced abortion in Yakutia

Currently, induced abortion is the only form of birth control covered by the compulsory medical insurance system of the Russian Federation. This helps to explain the increase seen in infertility and pregnancy complications due to implantation problems from endometrial irregularities (67). Indeed, within 6 months of their sexual debut, 28–46% of young Russian women undergo induced abortion (5).

In Yakutia, the trend in induced abortion is shown in Table 1.3. Some 5.4% of women of reproductive age have undergone induced abortion during the period 2004–2008, compared to 3.6% in the Russian Federation as a whole.

In the Russian Federation in 2002, 40.6 abortions were performed per 1,000 women of reproductive age. In Yakutia, this figure was 60.9 per 1,000 women of reproductive age. In 2008, this decreased to 31.2 per 1,000 women of reproductive age in the Russian Federation as a whole, and to 48 per 1,000 women of reproductive age in Yakutia.

Teenage pregnancy continues to be a problem. Most teenage pregnancies are unplanned, and as a result, unwanted. Information on contraception is not easily accessible to teenagers. The stigma associated with teenage pregnancy, and society's unwillingness to acknowledge this problem, are 2 factors which make abortion – often beyond the first trimester – an all-too-popular option for teenagers.

Among all abortions, the proportion of spontaneous abortions has remained stable. In 2008 they accounted for 10%, whereas induced abortion before 12 weeks accounted for 87% and induced abortion after 12 weeks 2.4%.

In studying the ratio of induced abortions to births over the period 2004–2008, induced abortions exceeded births until 2007, when the trend was reversed. However, the abortion rate continues to be too high to correct the serious demographic situation in Yakutia. During this period, the proportion of pregnancies terminated that were first pregnancies averaged 17%. In terms of age distribution, 85% were in women between the ages of 19 and 30, 15% were in women aged 31–35.

### 7.2. Reproductive health in teenage girls and women of reproductive age following induced abortion

The physiological repercussions of abortion can have a lasting impact on a young woman's health. This includes chronic endometritis, often of autoimmune origin, leading to infertility, and also to a variety of pathological processes of the uterus and other reproductive organs. Persistent endometritis impedes implantation of the fertilized ovum, subsequent embryogenesis and can lead to a range of pregnancy and labour complications. This diminishes the reproductive capacity of our country (74).

We attempted to evaluate the effectiveness of a variety of treatments aimed at restoring reproductive health in 73 teenage girls and 87 women of reproductive age following the artificial termination of a first pregnancy. This included:

- Comprehensive anti-inflammatory therapy including antimicrobial therapy.
- Hormonal contraception – use of low-dose oral contraceptives beginning immediately following the abortion, for a minimum of 6 months.
- Restoration of the natural flora of the digestive tract and vagina.
- Extensive rehabilitative therapy.

In our study, we used a combination of low-dose alternating magnetic field therapy, sinusoidal modulated current and intravaginal introduction of hyaluronidase and dimexidum. Prior to the therapy session, a tampon with hyaluronidase and dimexidum was inserted into the vagina. Then, magnetic therapy was carried out with the Magniter AMT-02 device using low-frequency alternating magnetic currents. Sinusoidal current #5 was used, then pulse current #5. Magnetic field intensity – 30mT, exposure time 15 minutes.

Immediately following the administration of alternating magnetic current, sinusoidal modulator current was given in stimulatory mode, alternating current mode (P1), operating mode (PP), frequency at 50 MHz, depth of modulation 100%, strength of current until contraction of anterior abdominal muscles, and time of exposure 10 minutes. This was carried out 10 times per day. Magnetic field therapy was chosen for its anti-inflammatory and pro-circulatory effects, and also lack of thermal effects, which makes it possible to use this form of therapy as soon as 2 hours after pregnancy termination. The therapeutic effects of sinusoidal modulated current are from its uterotonic, anti-inflammatory and anti-fibrotic effects, and also its pro-trophic effects on tissue (101).

Women who had undergone induced abortion were divided into 2 groups: those who had received our prescribed therapy, and those who had not. The allocation was as follows (Table 7.1).

**Table 7.1:** Allocation of subjects for post-abortion magnetotherapy

Group		Total	Treatment	No treatment
Russians	Teenage girls	19	11	8
	Women of reproductive age	24	13	11
Yakuts	Teenage girls	25	13	12
	Women of reproductive age	33	18	15
Evenks	Teenage girls	29	14	15
	Women of reproductive age	30	15	15

We measured involution of the uterus at 3, 6 and 8 days after pregnancy termination (Tables 7.2–7.4).

**Table 7.2:** Size of uterus 3 days following pregnancy termination

				Uterine dimensions (mm)		
				
Group			n	Length	Antero-posterior diameter	Width
Russians	Girls	Therapy	11	68.1±0.43*	57.1±0.38*	58.1±0.32*
		No therapy	8	71.1±0.43	60.1±0.44	60.1±0.43
	Women	Therapy	13	70.4±0.46*	58.8±0.34	60.9±0.36
		No therapy	11	73.4±0.48	60.4±0.44	61.1±0.41
Yakuts	Girls	Therapy	13	67.7±0.53	57.9±0.38	58.9±0.39
		No therapy	12	69.8±0.51*	60.8±0.44*	60.8±0.44
	Women	Therapy	18	68.7±0.53	60.1±0.44	60.7±0.44
		No therapy	15	72.7±0.50*	63.1±0.47*	63.1±0.47*
Evenks	Girls	Therapy	14	69.7±0.44	59.1±0.45	59.1±0.45
		No therapy	15	71.0±0.41	60.6±0.48	60.6±0.48
	Women	Therapy	15	67.7±0.53	58.3±0.47	58.3±0.47
		No therapy	15	77.1±0.61*	62.1±0.47*	62.1±0.47*

* Note: *p<0.05 – a statistically significant difference was found compared to the no therapy group.

**Table 7.3:** Size of uterus 6 days following pregnancy termination

				Uterine dimensions, mm		
				
Group			n	Length	Antero-posterior diameter	Width
Russians	Girls	Therapy	11	58.1±0.43*	48.1±0.38*	50.1±0.32*
		No therapy	8	59.1±0.43	54.1±0.44	56.1±0.43
	Women	Therapy	13	60.4±0.41*	54.8±0.34	50.9±0.36
		No therapy	11	67.4±0.48	58.4±0.44	60.1±0.41
Yakuts	Girls	Therapy	13	54.7±0.53	57.9±0.38	58.9±0.39
		No therapy	12	59.8±0.51*	60.8±0.44*	60.8±0.44
	Women	Therapy	18	53.7±0.59	53.1±0.44	50.7±0.44
		No therapy	15	62.7±0.50*	63.1±0.47*	53.1±0.47*
Evenks	Girls	Therapy	14	49.7±0.44*	54.1±0.45	49.1±0.45
		No therapy	15	61.0±0.41	60.6±0.48	53.6±0.48
	Women	Therapy	15	57.7±0.53	56.3±0.47	55.3±0.47
		No therapy	15	69.1±0.61*	62.1±0.47*	62.1±0.47*

* Note: *p<0.05 – a statistically significant difference was found compared to the no therapy group.

**Table 7.4:** Size of uterus 8 days following pregnancy termination

				Uterine dimensions, mm		
				
Group			n	Length	Antero-posterior diameter	Width
Russians	Girls	Therapy	11	47.1±0.33*	46.1±0.3*	44.1±0.32
		No therapy	8	57.1±0.32	52.1±0.24*	49.1±0.3
	Women	Therapy	13	48.9±0.23*	47.8±0.24*	50.9±0.27*
		No therapy	11	60.3±0.28	50.4±0.3	54.1±0.25
Yakuts	Girls	Therapy	13	47.6±0.33*	46.1±0.3*	44.1±0.32
		No therapy	12	57.1±0.32	53.1±0.21*	48.4±0.3
	Women	Therapy	18	49.9±0.23*	47.5±0.24*	50.0±0.23*
		No therapy	15	60.6±0.28	50.8±0.3	54.1±0.21
Evenks	Girls	Therapy	14	47.6±0.33*	46.1±0.3*	44.1±0.32
		No therapy	15	57.1±0.32	53.1±0.21*	48.4±0.3
	Women	Therapy	15	49.7±0.23*	47.5±0.24*	50.0±0.23*
		No therapy	15	61.0±0.22	51.3±0.3	53.7±0.22

* Note: *p<0.05 – a statistically significant difference was found compared to the no therapy group.

On the third day following pregnancy termination (Table 7.2), the size of the uterus in teenage girls and women of reproductive age undergoing therapy was significantly smaller compared with the group not receiving therapy. This difference was seen in all 3 ethnic groups. In teenage girls, a significant difference was observed in all uterine dimensions, and in women of reproductive age a significant difference was observed only in uterine length. The decrease in uterine dimensions was significantly more pronounced in teenage girls compared with women of reproductive age, which could be due to increased contractility of the myometrium in that age group.

On the sixth day following pregnancy termination, a decrease in all measured dimensions was observed compared with the third day (Table 7.3). The tendency for more pronounced contraction of the uterus in girls and women receiving therapy continued to be observed on the sixth day.

On the eighth day following pregnancy termination, uterine dimensions in the group receiving therapy were indistinguishable from a non-pregnant uterus (Table 7.4).

**Table 7.5:** Serum levels of FSH (IU/L), LH (IU/L) and prolactin (IU/L), early follicular phase

Group			n	FSH	LH	Prolactin
Russians	Girls	Therapy	11	3.0±1.4	4.2±1.1	206.4±41.5
		No therapy	8	3.7±1.6	9.2±1.1*	342.4±41.5
	Women	Therapy	13	5.4±1.2	6.1±1.3*	417.8±280.1
		No therapy	11	6.1±2.0	11.1±3.8*	607.8±240.1*
Yakuts	Girls	Therapy	13	7.0±3.4*	4.2±1.1	216.6±71.5
		No therapy	12	5.0±3.0	12.2±1.9*	376.9±81.9
	Women	Therapy	18	8.1±2.0*	5.1±2.6	287.8±190.7
		No therapy	15	11.1±2.9*	6.1±3.8	307.8±240.1
Evenks	Girls	Therapy	14	3.0±1.4*	4.2±1.1*	206.4±41.5
		No therapy	15	5.1±1.7	15.2±3.5	304.7±39.5
	Women	Therapy	15	6.8±4.9	7.1±2.4	337.8±170.8
		No therapy	15	10.8±4.9*	6.1±2.8	437.8±140.3

* Note: *p<0.05 – a statistically significant difference was found between therapy and no therapy group.

On the eighth day following pregnancy termination, a statistically significant difference in all parameters was seen between the women receiving therapy and those who received no therapy. In women not receiving therapy, uterine dimensions remained expanded.

In teenage girls receiving therapy, not 1 case of endometritis was diagnosed, compared to 20% of teenage girls not receiving therapy. No statistically significant difference was observed between ethnic groups. In women of reproductive age receiving therapy, acute endometritis was diagnosed in 11% of patients, compared to 27% among women not receiving therapy. Among the women who received post-abortion therapy but developed endometritis, all had a history of chronic salpingo-oophoritis. It is evident that induced abortion was a factor in precipitating a flare-up of chronic adnexal inflammation resulting in endometritis. This post-abortion treatment helped decrease the frequency of complications such as post-abortion endometritis by a factor of 2.

We studied hormone levels in participants not taking oral contraceptives at 1, 3 and 6 months following pregnancy termination. We found that in teenage girls, FSH levels returned to normal by 6 months following pregnancy termination, significantly later than women of reproductive age, whose FSH levels returned to normal within 3 months of pregnancy termination. In addition, teenage girls were found to have increased FSH levels compared to women of reproductive age at 1 and 3 months following the procedure. Teenage girls and women of reproductive age from the Yakut and Evenk groups were also found to have higher FSH levels compared to the Russian group.

We found that LH levels were elevated 1 month following pregnancy termination in the teenage girl group. Average LH levels had decreased by the third month following pregnancy termination. Among women of reproductive age, no LH surge was observed.

At 6 months following pregnancy termination, the average LH:FSH ratio in teenage girls was 2.3: 1; in women of reproductive age it was significantly lower, 1.1: 1. Our data shows that, in teenage girls who are still undergoing maturation of hypothalamo–hypophyseal structures and ovaries, induced abortion leads to polycystic ovarian syndrome in 1 in 5 girls; significantly more often (2.7 times) than observed in women of reproductive age.

PRL levels on days 2–3 of the menstrual cycle returned to normal in the teenage girl group by 6 months following pregnancy termination. In women of reproductive age 3 months following pregnancy termination, PRL levels remained moderately elevated in 87% of patients, compared to 48% of teenage girls. No differences were found among ethnic groups. PRL levels returned to normal in all teenage girls by 6 months following pregnancy termination, but remained moderately elevated in 20% of women of reproductive age.

Using progesterone levels measured on days 22–24 of the menstrual cycle as a marker for ovulation, we found that ovulation was restored by the third month following pregnancy termination. In 22% of teenage girls, progesterone levels above 20 nmol/L were observed. The proportion was significantly greater in women of reproductive age (47%).

Among teenage girls, induced abortion was found to lead to polycystic ovarian syndrome in 10% and anovulatory menstruation in 56% of patients. In women of reproductive age, termination of first pregnancy was found to lead to different types of hormonal disturbances – hyperprolactinaemia was seen in one-fifth of women, and anovulatory menstruation in one-third.

In comparing hypothalamo–hypophyseal and ovarian function at 9 months following first pregnancy termination, teenage girls receiving our recommended therapy had significantly lower FSH and LH levels than those not receiving our therapy (Table 7.5).

In terms of LH:FSH ratio, girls receiving therapy had an average LH:FSH ratio of 1.4: 1, compared to 2.68: 1 in girls not receiving therapy. Not 1 case of polycystic ovarian syndrome was diagnosed among the teenage girls receiving our therapy following pregnancy termination, compared with 11% of teenage girls not receiving therapy.

Girls not receiving our therapy had higher PRL levels than girls receiving our therapy. However, in neither group was the average PRL level outside normal ranges. Elevated PRL levels were observed in women of reproductive age, this was particularly evident among Russians.

No major change in progesterone levels during the luteal phase was observed between 6 and 9 months following pregnancy termination in either of the age groups.

On the whole, these results show that our therapy helped restore a biphasic menstrual cycle in the overwhelming majority of patients and decreased the risk of menstrual disturbances caused by polycystic ovarian syndrome and hyperprolactinaemia. However, we want to emphasize that a randomized controlled trial is the preferred method for testing the efficacy of our proposed therapy.

A number of statistically significant, ethnicity-dependent differences were also found in the post-abortion period. We found that artificial termination of a first pregnancy most often resulted in:

- Chronic inflammatory processes of the uterus and adnexa in Evenk women.
- Polycystic ovarian syndrome in teenage girls of Russian ethnic origin.
- Hyperprolactinaemia in women of Russian ethnic origin.

We measured levels of embryotropic autoantibodies 3 weeks following cessation of any medical therapy. We found that all teenage girls who had received hormonal and rehabilitation therapy during the post-abortion period exhibited normoreactivity based on embryotropic autoantibody level. In the Yakut and Russian groups, fewer girls (by a factor of 1.3) exhibited normoreactivity, but no statistically significant difference was found with the group receiving therapy. Significantly fewer (1.5 times) Evenk girls who did not receive therapy, however, were found to be normoreactive.

A similar situation was found among women of reproductive age – on the whole, our post-abortion therapy aided in normalization of embryotropic autoantibodies. Further analysis showed that the majority of the girls and women diagnosed with hyporeactivity also exhibited signs of acute post-abortion endometritis. Hyperreactivity was found in girls and women with polycystic ovarian syndrome and hyperprolactinaemia.

Teenage girls from the Russian group were more negatively affected by induced abortion, evidenced by a significantly higher prevalence of menstrual disturbances due to polycystic ovarian syndrome following termination of their first pregnancy. Cases of inflammatory processes of the uterus and adnexa were evenly distributed among ethnic groups.

Variation in health impacts of pregnancy termination by ethnicity was most pronounced among women of reproductive age. In the Russian group, hyperprolactinaemia, anovulatory menstruation and luteal phase defect were the most common complications. Evenk women experienced inflammation of the uterus and adnexa most often. Despite the fact that we cannot make judgments about which groups tolerated induced abortion “better,” we would like to note that Yakut women had the fewest complications during the post-abortion period compared with women from the other 2 ethnic groups. This was observed in terms of inflammatory processes of the uterus and adnexa and hormonal disturbances, as well as in the immunoreactivity studies (more Yakut women were normoreactive).

It is our hope that the results of this study will serve to underscore the need to preserve the reproductive health of teenagers in the Far North. This area is plagued by a high prevalence of gynaecologic disorders and sexually transmitted infections, as well as inadequate awareness about sex and reproduction.

The teenage years are a time for rebellion and experimentation, and teenagers are known for their impulsivity. This often gets in the way of rational decision-making and careful consideration of consequences, which so often leads to risky sexual behaviour, unwanted pregnancies, and transmission of sexually transmitted infections. The following should be implemented at the government level in order to promote responsible sexual behaviour and prevent life-altering consequences:

- School and community awareness-raising programs aimed at promoting responsible sexual behaviour.
- Creation of resource centres with involvement of gynaecologists, contraception specialists, psychologists, pedagogues and addiction counsellors.
- A program making possible yearly, comprehensive physical examinations for girls of all ages. Reproductive behaviour will be taken into consideration, allowing for early detection of potential problems.
- Cessation of surgical abortions in teenagers in favour of medical abortions with subsequent treatment aimed at preserving reproductive function.

## Conclusions

Russia is at a point in its development where preservation of reproductive health is essential not only for demographic security, but also national security (102). Reproductive health ensures a country's future demographic prosperity. One of the goals of Russia's current demographic policy is to “strengthen the reproductive health of the population” (103). However, federal legislation does not currently provide for the conditions that would make this possible (104).

Worldwide, more than 46 million pregnancies end in abortion every year (105). In Russia, somewhere between 11 and 40%, or more, of first pregnancies end in abortion (106). The absolute number of abortions is decreasing, and the ratio of births-to-abortions has improved to somewhere between 1: 0.9 (official statistics) and 1: 1.6–1.7 (expert estimates). Nevertheless, induced abortion is diminishing the reproductive capacity of our country.

Among those women whose fertility remains intact following an abortion, there is nevertheless a greater risk for chronic, often autoimmune, endometritis to which future pregnancies would be subject. Endometritis puts a women more at risk for infertility, implantation defects and miscarriage. A pregnancy conceived against a background of endometritis is at a greater risk for intrauterine growth retardation, perinatal morbidity and mortality (106). In Russia, between 3 and 4 million women of reproductive age suffer from infertility. Of these, 25% require in-vitro fertilization to conceive (107).

Russia is a country of many ethnicities, living in a variety of social contexts, under diverse climatic and environmental circumstances. This diversity is reflected in variations in child development observed in different regions. The reproductive health of Russian girls today is a factor with the potential to affect the health of the next generation of Russians. Many regions have an extremely high prevalence of gynaecologic disorders in their girls, and the official statistics do not reflect the actual situation. By some estimates, more complete physical examinations could increase the current figure by a factor of more than 10 (108). A girl's physical development is one of the most important indicators of her health, and this is closely associated with the development of the reproductive system.

As an objective indicator of body composition, we measured height and weight. By weight, all groups were equivalent. However, teenage girls and women of Russian ethnicity were, on average, significantly taller compared with the indigenous groups. No statistically significant difference was found between urban and rural dwellers in terms of underweight. A different situation was seen in Evenk girls. Underweight girls have been shown to have an increased risk of experiencing reproductive problems in the future (109). Significantly fewer girls were found to be overweight, in both urban and rural areas.

Pelvimetry revealed an increase in all 4 external pelvic measurements by the age of 10–11. At this age, all young girls of Yakut and Evenk origin began experiencing significant pelvic growth. The next increase in all transverse dimensions was observed at the beginning of the late pre-pubertal period. By 17 years of age, the overwhelming majority of young girls of Yakut and Evenk origin ceases to experience pelvic growth, in contrast to 17-year-old Russian girls. The average pelvic size was 0.5–1.2 cm smaller in girls of Russian ethnicity. According to Kokolina, girls in the Russian Federation as a whole experience most of their pelvic growth by age 14 (110).

In examining development of secondary sex characteristics by age, we found that level of sexual development was highly variable at age 13. Precocious development was observed equally among urban dwellers and rural dwellers. However, delayed maturation was observed significantly more often in rural dwellers. No difference between groups was observed in the order of appearance of secondary sex characteristics. Fewer girls living in rural areas had completed puberty by age 17 compared to urban dwellers. By starting puberty at a later age, we found that Evenk and Yakut girls experienced more rapid breast development.

When examining the main anthropometric parameters, Yakuts and Evenks are similar to other indigenous peoples of the North, Siberia and the Far East. These groups are, in general, characterized by short stature and stockiness attributable to harsh climate and other region-specific conditions.

The average age of menarche in the Russian group was 12.6±1.1 years, compared to 13.8±0.99 years in Yakuts and 13.9±0.9 years in Evenks. The menstrual cycle became established either immediately or within the first 6 months among fewer than half of girls in all ethnic groups. The analysis showed that girls today take longer to establish a regular menstrual cycle. According to a study in Moscow, 18% of teenage girls have irregular menstrual cycles during the second year of menstruation (111).

Teenage girls living in Yakutia, regardless of ethnic origin, can be characterized by early sexual debut and a high frequency of poor health habits such as smoking and alcohol consumption. The great majority used abortion as the method of contraception. Currently, women who have never given birth are not recommended to utilize an intrauterine device, nor are women who are at a greater risk for sexually transmitted infections (112, 113). Women who have not given birth who utilize intrauterine devices are at a higher risk for bacterial vaginosis, *Urealyticum*, *Mycoplasma hominis* and various forms of candidiasis (114). Various authors, on the other hand, consider intrauterine devices acceptable for teenagers, especially levonorgestrel-containing devices (115–117).

Our research found that only 1 in 3 women of reproductive age in Yakutia is currently married. This reflects the situation in the country as a whole, where there is a growing trend among women and men to avoid marriage (118). Currently, Russia is experiencing a radical, unprecedented change in marriage behaviour, with a sharp increase in the age of first marriage. Among younger people, early marriage is viewed as interfering with personal success. The dynamic we are seeing in marriage in Russia is part of a growing crisis of the family as a social institution.

Despite the fact that more than half of all Evenk women are married, we did not observe a tendency towards use of highly effective contraceptives. According to a study in China, among rural married couples using condoms as the primary form of birth control, the abortion rate was 5.5%, compared with 2.4% in married couples using an intrauterine device (119).

Many studies have demonstrated a decrease in the age of sexual debut, multiple sexual partners and an increased tendency to resort to abortion, and yet lacking in knowledge of contraception and preventive sexual health practices (120). Lack of a trusting relationship between the girl and her mother, and having a mother with a favourable attitude towards sexual activity at a young age have been found to be predictors of more sexual partners in young and teenage girls (121).

Girls in Yakutia generally lack information about family planning, sexuality and modern methods of contraception, similar to findings from other studies (122). Increasingly, the Internet should be considered an important source of information for teenage girls (123). One solution is to provide girls with prompt, anonymous advice from a gynaecologist free-of-charge.

Known risk factors for teenage pregnancy include early sexual debut, low socio-economic status and family disruption (a broken family or spending long periods without both parents) (124). Studies conducted both in Russia (4, 125) and abroad (126) have found that the reproductive health of teenage girls who use psychoactive substances can be characterized by early sexual debut, a high rate of sexual activity, frequently changing sexual partners and inadequate knowledge about contraceptive methods (4, 125). Use of alcohol and tobacco and early sexual debut are prognostic indicators of suicidal thoughts and suicide attempts during the reproductive period (126).

Improving maternal health is difficult to accomplish without addressing the problems of unsafe abortions and associated morbidity and mortality (127). We are convinced that the deterioration of reproductive health in girls and teenage girls in Yakutia mandates the mobilization of government resources in order to bring down the level of both gynaecologic and non-gynaecologic disorders and prevent teenage pregnancy and abortion. The creation of centres for providing contraceptive services and high-quality women's health care will help to lower the rate of infertility, and complications from pregnancy and labour in the near future.

The proportion of teenage pregnancy that is unwanted, the proportion that is carried to term, and the frequency of repeat abortions vary considerably across studies (105),^128^–^130^). In Yakutia, among women of reproductive age of any group, only 1 in 2 pregnancies resulted in a birth. Women of reproductive age from the indigenous groups had more births than Russian women, and also more early pregnancy terminations. A phenomenon such as abortion is dependent, to a large extent, on breaking “social habits” and not just preventing unwanted pregnancy (131). The emotional consequences of abortion have been well documented, especially in older women and women who are already mothers (132).

Results of our microscopic and bacteriologic studies are consistent with others, showing an excess of micro flora in the vaginal secretions of pre-pubertal girls (133). Elsewhere in Russia, girls suffering from inflammatory processes of the vulva and vagina, about a third had bacterial vaginosis (134). The high frequency of candidal vulvovaginitis among the women we examined agrees with other studies. Candidiasis can be considered a marker of ill health in a woman's body as whole (135–137). Bacterial vaginosis is among the most significant non-communicable diseases in women (138, 139). Women with a history of sexually transmitted infections or pelvic inflammatory disorders are at a higher risk for ectopic pregnancy (140).

The outcome of a woman's first pregnancy is an important indicator of both reproductive behaviour and reproductive health. A pregnancy with an unfavourable outcome, or a complicated course, is an indicator of a woman's subsequent health, reproductive function, and the physical and reproductive health of her offspring (141). Women of reproductive age who take oral contraceptives experience positive changes in their physical and reproductive health, including: less blood flow to the reproductive organs, more regular menstrual cycles, more regular autonomic nervous system, a lower risk of ischemic heart disease over 10 years (142, 143).

Researchers have recently been particularly interested in rehabilitation of the endometrium. Rehabilitation is considered to be therapy aimed at restoring endometrial function, which could be diminished following any intrauterine disturbance (144). Women with recurrent miscarriage should be treated with an individualized pregnancy preparation protocol and regularly monitored. Also, planning of subsequent pregnancies should be carried out (145).

We set out to evaluate the effectiveness of a program to restore the reproductive health of a sample of teenage girls and women who had undergone artificial termination of their first pregnancy. This included:

- Comprehensive anti-inflammatory therapy including antimicrobial therapy.
- Hormonal contraception – use of low-dose oral contraceptives beginning immediately following the abortion, for a minimum of 6 months.
- Restoration of the natural flora of the digestive tract and vagina.
- Extensive use of physical therapy.

In our study, we used a combination of low-dose alternating magnetic field therapy, sinusoidal modulated current and intravaginal introduction of hyaluronidase and dimexidum. Magnetic field therapy was chosen for its anti-inflammatory and pro-circulatory effects, and also lack of thermal effects, which makes it possible to utilize this form therapy as soon as 2 hours after the pregnancy termination procedure. The therapeutic effects of sinusoidal modulated current are from its uterotonic, anti-inflammatory and anti-fibrotic effects, and also its pro-trophic effects on tissue (101).

Many researchers have noted the benefits of rehabilitation procedures on the endometrium (146), including magnetic field therapy on restoring the receptivity of the endometrium in cases of endometritis (147, 148). The vasodilatory effects of magnetic field therapy helped increase the effectiveness of in-vitro fertilization and decrease the rate of pregnancy loss (147). Others have also proposed magnetic field therapy as an alternative treatment in young girls with recurrent bacterial vaginosis (149).

As yet the therapy has not undergone evaluation with a randomized controlled trial regarding its efficacy. Such a study is needed before it can be recommended widely.

Our study showed that artificial termination of a first pregnancy led to a variety of conditions including post-abortion endometritis, polycystic ovarian syndrome, anovulatory menstruation, luteal phase defect, and immunoreactivity disturbances. Anovulation and luteal phase defect occurred as a result of polycystic ovarian syndrome or hyperprolactinaemia (150, 151). Polycystic ovarian syndrome is often seen with metabolic disturbances such as obesity and hyperinsulinaemia (152). Women with hyperprolactinaemia suffer from menstrual irregularities (153), the primary gynaecologic disturbance being anovulation, which can be associated with oestrogen secretion disturbances brought about by either high or low FSH levels (154), although many cases are idiopathic (155).

It is our opinion that pregnancy termination leads to serious hormonal disruption, initiating a chain of pathologic events, particularly in the functionally and morphologically immature hypothalamo–hypophyseal–ovarian system. Others share this view, and consider initiation of oral contraception immediately following a surgical or medical abortion warranted to prevent undesirable reproductive consequences (156).

It is our hope that the results of this study will serve to underscore the need to preserve the reproductive health of teenagers in the Far North. The following should be implemented at the government level in order to promote responsible sexual behaviour and prevent life-altering consequences:

- School and community awareness-raising programs aimed at promoting responsible sexual behaviour.
- Creation of resource centres with involvement of gynaecologists, contraception specialists, psychologists, pedagogues and addiction counsellors.

The formation of appropriate attitudes and behaviours surrounding reproduction should be based on educational programs promoting a self-preservation concept of reproductive health. Such programmes should be incorporated into the study plans of secondary schools, specialized secondary schools and institutions of higher education.

In order to ensure early detection of risks to reproductive health, a program should be implemented making possible yearly, comprehensive physical examinations for girls of all ages. More first-time pregnancy terminations should be carried out using medical, not surgical, means, with immediate post-procedure rehabilitation. Rehabilitation following pregnancy termination should include comprehensive medical and physical therapy.

Since 2011, a 3-tiered system has been in place for provision of medical assistance to women during pregnancy, delivery and in the post-partum period in Yakutia. The first tier encompasses 31 birthing departments, the second–6 birthing departments, and the third–a perinatal centre. A distance midwife consultation centre has been created which is responsible for routine and emergency monitoring of pregnant women. Three interregional birthing departments were opened in the regional hospitals of Megino-Kangalasskaya, Verkhoyansko and Vilyuyskaya. Two more interregional birthing departments are planned, in Mirninskaya and Srednekolymskaya. There are 40 beds for neonatal intensive care. In 2011, a mammology centre was opened at the Republic Hospital #1. It is responsible for breast disorders and managing the mammology service.

Three new standards have been implemented to improve the care of pregnant women to ensure high-quality care for post-partum haemorrhage, pre-term births and pregnancy-induced hypertension with proteinuria. The latest in birthing methods have also been implemented in our regional birthing departments, including home births, partner births, joint infant–mother rooms, early introduction to the breast, prioritization of breast feeding and prevention of hypothermia in the newborns. In 2011, a medical–psychological service was established aimed at decreasing the number of abortions and preventing the relinquishment of parental rights.

In 2011, Yakutia was included in a group of regions carrying out activities aimed at diagnosing prenatal developmental disturbances, as part of the Wellness National Priority project. Under this program, every pregnant woman will be given an ultrasound with biochemical screening. As part of the sub-program *Families and Children of Yakutia: Family*, physicians travelled to Tomponsky, Ust-Aldansky, Namsky, Bulunsky, Anabarsky and Allaikhovsky regions to provide physical examinations for women. All pregnant women underwent ultrasonography.

In 2011, birthing departments received new equipment under the following programmes: the Public Health Modernization Program, Protecting the Health of Yakutia: Protecting the Health of Women and Children, the Wellness Program and the Prenatal Diagnostics Program.

It is evident that substantial progress in reproductive health has already taken place in Yakutia. It is hoped that comprehensive research such as ours will provide the evidence needed to design more effective programs and services.
